# The Role of Enteral Nutrition in Patients with Inflammatory Bowel Disease: Current Aspects

**DOI:** 10.1155/2015/197167

**Published:** 2015-02-22

**Authors:** John K. Triantafillidis, Costas Vagianos, Apostolos E. Papalois

**Affiliations:** ^1^Inflammatory Bowel Disease Unit, IASO General Hospital, 264 Mesogeion Avenue, Holargos, 15562 Athens, Greece; ^2^1st Surgical Unit, Saint Panteleimon Hospital, D. Mantouvalou 3, 18454 Nicea, Greece; ^3^Experimental-Research Center, ELPEN Pharmaceuticals, 95 Marathonos Avenue, Pikermi, 19009 Athens, Greece

## Abstract

Enteral nutrition (EN) is considered to be of great importance in patients with inflammatory bowel disease (IBD) and nutritional problems. This comprehensive review is aiming to provide the reader with an update on the role of EN in IBD patients. EN can reduce Crohn's disease (CD) activity and maintain remission in both adults and children. Nutritional support using liquid formulas should be considered for CD patients and in serious cases of ulcerative colitis (UC), especially for those who may require prolonged cycles of corticosteroids. Given that the ultimate goal in the treatment of CD is mucosal healing, this advantage of EN over corticosteroid treatment is valuable in therapeutic decision-making. EN is indicated in active CD, in cases of steroid intolerance, in patient's refusal of steroids, in combination with steroids in undernourished individuals, and in patients with an inflammatory stenosis of the small intestine. No differences between the efficiency of elemental diets and nonelemental formulas have been noticed. EN must be the first choice compared to TPN. EN has a restricted value in the treatment of patients with large bowel CD. In conclusion, it seems important not to underestimate the role of nutrition as supportive care in patients with IBD.

## 1. Introduction

Nutritional disturbances represent a frequent manifestation of inflammatory bowel disease (IBD) patients, especially those with Crohn's disease (CD). Despite the fact that patients with ulcerative colitis (UC) in remission usually exhibit normal nutritional status, this can be rapidly deteriorated during the active phase of the disease. According to recently released guidelines by the Austrian Working Group on Nutrition and IBD [1] and the British Dietetic Association [2], the existence of malnutrition should always be assessed in patients with IBD and weight loss (>* *5* *% within 3 months) and nutritional deficiencies or after extensive bowel resection [1], while enteral nutrition (EN) either elemental or nonelemental could be offered as an alternative option to induce disease remission [2]. These very recently published guidelines underline the importance of applying EN in patients with CD and the reemersion of scientific interest in the field of nutrition in IBD. Channeling the scientific community's interest towards cheaper and safer treatment strategies, such as EN, was significantly promoted by the bad economic situation and the continuously rising cost of treatment of IBD patients.

The aim of this comprehensive review was to provide the reader with an update on the role of EN in IBD patients based on the results of past and current relevant literature.

## 2. Methodology

A comprehensive computerized literature search strategy, having used the electronic database search engines CINAHL, Cochrane Library, EMBASE, MEDLINE, Scopus, and Web of Science up to May of 2014, was implemented. The medical subject headings applied were “enteral nutrition,” “nutrition,” “inflammatory bowel disease,” “Crohn's disease,” and “ulcerative colitis.” All full-length randomized, double-blind, randomized placebo-controlled, or controlled against a conventional treatment clinical studies were included in the analysis. Then, studies deemed eligible for inclusion were manually searched. The studies were divided into those that have assessed the use of EN for the induction of remission and maintenance of remission in UC and those referring to the induction of remission and prevention from operative recurrence for CD. Data collection included the authors' names, year of publication, number of subjects included in the study, types of EN administered, treatment duration, length of follow-up, remission and response rates, and adverse effects.

Studies dealing with the nutritional status and the causes of malnutrition in patients with IBD were also included in order for the reader to have a more spherical view of the subject of nutritional therapy of patients with IBD.

From 1993 to 2014, we have identified 34 clinical studies dealing with the treatment of IBD with EN in adults. There were 32 studies of EN therapy in CD and 2 studies in UC, including more than 1600 individuals in total.

## 3. Results

### 3.1. Nutritional Status in Patients with IBD

The nutritional status in patients with IBD is of paramount clinical importance. It is well established that nutritional deficits are related to the activity, location, and extent of the disease. It has been estimated that 23% of IBD patients, who are regularly followed up in the outpatient clinic, as well as 85% of hospitalized IBD patients have nutritional disturbances. Differences are seen between patients in an active phase and those in remission. Weight loss is found in up to 75% of hospitalized patients with an exacerbation of CD, with a negative nitrogen balance present in more than 50% [3, 4].

The most prevalent form of malnutrition in CD patients is an excess of body weight, concomitantly with an inadequate dietary intake, namely, micronutrients, clearly related to dietary exclusion of certain foods. Patients with small bowel CD usually have more prominent nutritional deficits compared with patients without small bowel involvement.

Reduced food consumption remains the main cause of poor nutritional status. Its etiology is related to the presence of anorexia because of the chronic inflammatory process, presence of vomiting, and dietetic limitations suggested by either the physician or the patient. Other important factors include reduced absorption from the inflamed mucosa, reduced bowel length due to surgical resections, and increased losses from the inflamed mucosa [5–8]. Bypass of healthy mucosa due to surgical interventions or fistulae formation could lead to malabsorption.

Various drugs administered for the treatment of the underlying IBD could be related to or could worsen the defective nutrient absorption. For instance, metronidazole frequently can cause dyspepsia, metallic taste, and reduced food consumption. Sulfasalazine reduces the absorption of folic acid and can cause hemolysis, while corticosteroids can impair calcium absorption and increase urine calcium excretion. Cholestyramine also can cause malabsorption of fat, calcium, and fat diluted vitamins.

Nutritional disturbances in patients with IBD include macro- and micronutrient deficiencies such as hypoproteinemia and hypoalbuminemia, electrolyte (calcium, magnesium, and potassium) and trace element (zinc, copper, and selenium) disturbances, vitamin (B12, A, complex B, C, D, and E) deficiencies, anemia (due to iron, B12, and folic acid deficiency), and loss of weight [9, 10]. Impaired nutritional status could influence both preoperative morbidity and mortality. Poor nutritional status can also impair healing of wounds and fistulae, as well as the ability of the patients to confront blood losses. It must be stressed that, although the assessment of the nutritional status of patients with IBD is important, it is frequently underestimated in everyday clinical practice.

The main causes of poor nutritional status in patients with IBD are shown in Table 1.

### 3.2. Mechanisms of Action of Nutritional Treatment in Patients with IBD

Malnutrition impairs negatively influences immune function and wound healing, having also negative as well as psychological and cognitive effects. Ensuring a good nutritional status would theoretically lead to enhanced mucosal healing [11].

The mechanism of action of EN and especially of elemental diet might be multifactorial [12]. Increased gut permeability is an important factor in the pathogenesis of IBD and elemental diet has been shown to decrease intestinal permeability [13]. It seems possible that many dietary antigens that induce inflammation could be avoided by elemental diet. Reduction in the workload of digestion and absorption by elemental diet and in peristalsis and digestive tract secretions may also play a role. Elemental diet might also reduce the amount of commensal gut bacteria participating in the development of inflammation in IBD.

It is possible that exclusive EN changes gut microbiota, although the exact pathophysiological role is unknown. EN has been shown to have an anti-inflammatory effect in children with CD [15]. Feng et al. showed that exclusive EN in patients with CD ameliorated mesenteric fat alterations by restoring normal morphology of adipocytes and reducing the production of proinflammatory cytokines in the mesenteric fat [16].

In conclusion, it seems that dietary components, bacteria, susceptibility genes, and the innate immune response are involved in the pathogenesis of CD, and EN has a significant impact on the cascade of pathogenesis [17, 18].

The main mechanisms, by which nutritional intervention can ameliorate the inflammatory activity in patients with IBD, are shown in Table 2.

## 4. Enteral Nutrition in Patients with IBD

EN has successfully been used as a supporting treatment in patients with IBD and nutritional problems. It has also been used as a primary treatment in patients with CD, although there is no consensus regarding its efficacy and exact indications. Patients with IBD must be supported with EN if oral intake is impossible or it is inadequate to restore the losses or malnutrition of nutrients.

EN has fewer side effects and is cheaper compared to TPN. Some problems could appear in patients receiving EN, mainly related to bad taste and use of catheters. Some patients on EN complain of colic abdominal pain, flatulence, and symptoms of gastroesophageal reflux. EN must be preferred to TPN in patients who have no indications of toxic megacolon, bowel obstruction, hemorrhage, and bowel perforation.

EN can be divided into elemental (containing only amino acids, glucose, and fatty acids, nutrients that do not need digestion), semielemental (containing only small peptides, oligosaccharides, and medium-chain fatty acids), and polymeric (containing proteins, carbohydrates, medium- and long-chain fatty acids, vitamins, and trace elements) [19]. Fiber is commonly added to polymeric feeds; however, there is little evidence to suggest that it has a substantial positive or negative effect on hospitalized patients [20].

Polymeric diets are most frequently used, while semielemental and elemental are used in patients with short bowel syndrome or patients who cannot tolerate polymeric diets [21]. We must bear in mind that a high amount of fat consisted of long-chain triglycerides in the elemental diet formula, which decreases its therapeutic efficacy against active CD [22].

### 4.1. Enteral Nutrition as a Primary Treatment in Patients with CD

The effectiveness of EN as a primary treatment in CD patients has been mainly studied during the previous two decades. Except for the restoration of nutritional deficits, EN can be used as a primary treatment in patients with CD.

EN for 3 to 6 weeks in patients with active CD reduces disease activity in 33–82% of them [23–25] although the results are less satisfactory compared to corticosteroids in most of studies including two meta-analyses [26, 27].

Table 3 shows the results of studies on the use of EN (elemental and polymeric diet) for the induction of remission in CD [28–30], while Table 4(a) shows the results of studies comparing elemental versus nonelemental diets in CD patients [31–33].

### 4.2. Elemental Diet versus Corticosteroids

Studies on the efficacy of elemental diets in CD patients have produced encouraging results.

Riordan et al. studied 136 patients with active CD who were treated exclusively with elemental diet for 2 weeks, though 31% of them did not tolerate the diet for more than 1 week. Of the 78 remaining patients, 84% achieved remission after a 14 d treatment course. Then, the group was split into 38 patients receiving a tapered course of prednisolone and advice on healthy eating and 40 patients receiving placebo instead of steroid. There was a median remission of 3.8 mo in the steroid group compared to 7.5 mo in the diet group [34].

In another study, 22 hospitalized patients with CD received elemental diet for 4 weeks, while 20 patients received prednisolone at a dose of 0.75 mg/kg daily for 2 weeks followed by reducing doses. Nine of the 22 patients (41%) in the diet arm withdrew because of intolerance. The reduction in disease activity was similar between the diet and prednisolone groups. Similar reductions in CRP and increases in serum albumin concentration were noticed in both groups. The probability ratio of remaining patients in remission was, however, much lower in the diet group. At 6 mos, this probability was 0.67 after steroid compared to 0.28 after elemental diet [35].

It seems that both EN and the chimeric anti-TNF antibody infliximab were associated with 61% and 70% reduction in endoscopic scores, significantly higher than corticosteroids and placebo, respectively. However, pooled results should be cautiously interpreted because of the diversity of measurements [36].

### 4.3. Polymeric Diet versus Corticosteroids

The use of a polymeric diet in adult patients with CD has also been studied. Seventeen patients with active CD received 1 mg/kg per day of prednisolone, followed by a reducing course, and 15 received polymeric diet, no medication, and no other food. Of the 17 patients in the CSs group, 15 entered remission after a mean time of 2 weeks. Of the 15 patients in the polymeric diet group, 12 entered remission after a mean time of 2.4 weeks. The cumulative probability of relapse during the follow-up period was higher in patients receiving CSs, although the differences were not statistically significant [37].

It has been suggested that the amount and type of fat in polymeric feeds may have an impact on its efficacy in CD patients. It seems that a polymeric diet rich in monounsaturated fatty acids (MUFA) would be more effective in inducing remission in active CD patients than an identical diet but with polyunsaturated fatty acid- (PUFA-) precursors of some inflammatory cytokines. Gassull et al. randomised 62 patients with active CD to either one of these diets for 4 weeks or to 1 mg/kg per day of prednisolone. The steroid group experienced a 79% remission rate while in the diet groups only 20% in the MUFA group and 52% in the PUFA group achieved this target. These results were quite the opposite of those expected [38].

In a randomised trial, 54 patients with active CD received a polymeric diet with either high or low long-chain triglyceride content. Of those completing the trial, the response rate was 46% for the low long-chain triglyceride group and 45% for the high long-chain triglyceride group, respectively, thereby demonstrating no significant difference in efficacy with differing fat composition [39]. Although EN is less effective than steroid therapy in this indication, it is recommended as a therapeutic alternative [40, 41].

Studies comparing EN with corticosteroids in patients with active CD are shown in Table 4(b).

### 4.4. Enteral Nutrition as a Maintenance Treatment for Crohn's Disease

So far, only few studies, aiming to evaluate the efficacy of EN as a maintenance treatment remission in CD patients, have been conducted. However, they all found a significant relapse-preventing effect, regardless of whether it was tested in patients with remission or not. This led to a positive evaluation of EN as maintenance therapy in a Cochrane review [42], although the authors criticized the limited number of prospective studies.

Verma et al. compared oral supplementation with elemental diet plus normal diet (first group) with unrestricted diet (second group) in a series of 39 patients with CD in clinical remission [43]. On an intention-to-treat basis 48% in the supplemented group remained in remission for 12 months, compared to 22% patients in the second group (*P* < 0.0003).

Takagi et al. applied a diet in which half of the daily calorie requirement was provided by an elemental diet and the remaining half by a free diet [44]. Fifty-one patients in remission were randomly assigned to a half elemental (*n* = 26) or a free diet group (*n* = 25). The relapse rate in the half elemental diet group was significantly lower (34.6% versus 64.0%) than that in the free diet group after a mean follow-up of 11.9 months.

Yamamoto et al. [45] in their study on the long-term efficacy of EN in patients with quiescent CD randomized their patients to receive nightly EN in addition to mesalazine as maintenance therapy, whereas the control group received mesalazine only. They found that patients on EN experienced significantly fewer clinical relapses, had less endoscopic disease activity, and finally had reduced levels of mucosal proinflammatory cytokines.

The same group of authors investigated the long-term effect of EN in a group of 40 patients who underwent resection for ileal or ileocolic CD. Following surgery, 20 patients received continuous elemental diet infusion during the nighttime plus a low-fat diet during the daytime (EN group), while 20 patients received neither EN nor food restriction (control group) for 5 years after operation. During the follow-up period of 5 years, 2 patients (10%) in the EN group and 9 (45%) in the control group developed recurrence (*P* = 0.03). The cumulative recurrence incidence rate requiring infliximab was significantly lower in the EN group compared with control group (*P* = 0.02). The cumulative incidence of reoperation was numerically lower in the EN group although difference did not reach statistical significance [46]. The results of this study suggest that EN reduces the incidence of postoperative CD recurrence.

Analysis of the studies devoted to EN reveals that their design has some common characteristics. The most important one is that patients were not on exclusive EN but used EN either as a supplement or as a nightly tube feeding in addition to their normal food. Therefore, the intake of normal food does not appear to have a negative effect. As a result, the reduction in antigen load might not play a significant role. The change in intestinal flora could be well a mechanism by which EN reduces inflammatory activity [47, 48].

Several studies showed that patients drinking more than 1200 kcal/day via EN fared better than those drinking less. In these studies, patients were free to eat a standard diet in addition to EN [49]. It has been suggested that the provision of easily absorbable substrates plays a crucial role in maintaining remission in CD [50].

In conclusion, it seems that partial EN might be an effective maintenance treatment for CD. The mechanism of action, as well as what type and what form of application of the diet might be optimal, is not clear. Further studies, investigating the effect of good-tasting oral EN that would increase total nutritional intake over that with a standard diet, must be conducted.

Table 5 shows results of prospective studies of EN as a maintenance treatment in patients with CD.

## 5. EN in Special Circumstances

### 5.1. EN and Concurrent Infliximab Administration

So far, 3 studies have investigated the efficacy of EN on the clinical course of patients with CD receiving biologic agents as a maintenance treatment. Two of them showed beneficial results. We suggest that this represents an interesting topic requiring further investigation.

In the first study, the efficacy of EN on the rate of clinical remission in patients with quiescent CD receiving infliximab was prospectively assessed [51]. Fifty-six patients who achieved clinical remission with infliximab continued to receive the biologic agent as maintenance treatment in a dose of 5 mg/kg every 8 weeks. Thirty-two of them received concomitant EN (elemental diet infusion during night-time and a low fat diet during daytime) (EN group), while the remaining 24 patients received neither nutritional therapy nor food restriction (non-EN group). During the follow-up period of 56-weeks CDAI did not significantly differ between the 2 groups. In an intention-to-treat analysis, no difference was noticed between the two groups suggesting that EN during infliximab maintenance therapy does not increase the rate of remission in patients with CD.

In another study aiming to elucidate factors other than infliximab promoting sustained response to infliximab in patients with CD, the authors noticed that, after a median follow-up of 85 weeks, concomitant use of EN therapy (elemental and/or polymeric formulas) was found to be an independent factor associated with sustained response to infliximab [52]. The authors suggested that concomitant use of EN more than 600 kcal/d is likely to yield a sustained response to infliximab in CD patients.

In the third study, patients with CD who achieved remission after triple infusions of infliximab followed by infliximab maintenance treatment every 8 weeks were classified into EN group and non-EN group (45 and 57 patients, resp.). The cumulative remission rate was significantly higher in the EN group than in the non-EN group (*P* = 0.009). Multivariate analysis revealed that EN was the only significant suppressive factor for disease recurrence (*P* = 0.01) [53]. So, the results of this study revealed that EN combined with infliximab maintenance treatment offered significant benefit on maintaining remission in patients with CD.

### 5.2. Influence of EN in the Quality of Life of Patients with CD

So far, two studies examined the effect of EN on the quality of life of patients with CD. Both studies showed significant improvement of the level of quality of life in those patients receiving EN.

The first study included 126 patients, 95 of whom received EN. Multiple regression analysis revealed that EN was an independent factor that improved the IBD Questionnaire total score in patients with disease duration of 10 years or more [54] suggesting that EN can improve the health-related quality of life of CD patients with long-term disease duration.

In the second study, 13 patients received a polymeric enteral feed in the daytime (orally) and via a nasogastric tube (at night) for 4 weeks. A significant improvement in the total IBD quality of life score (*P* < 0.001) was noticed with 11 patients (84.6%) achieving clinical remission [55]. Although the number of patients was small and a control group was absent, this study showed that a 4-week treatment with EN significantly improves health-related quality of life in adults with active CD.

### 5.3. Effect of EN on Hospitalization Rate

A relevant study investigated the effectiveness of EN in reducing the hospitalization rate in patients with CD. Among 135 out of 237 patients with ileal involvement who received EN providing 900 kcal/d or more a significant improvement in the cumulative nonhospitalization rate was noticed. On the contrary, among 31 patients without ileal involvement, the cumulative nonhospitalization rate did not differ among those receiving EN = or > than 900 kcal/d [56]. The authors concluded that the use of EN providing 900 kcal/d may be effective in avoiding hospitalization in CD patients with ileal involvement.

### 5.4. EN in Relieving Strictures in Patients with CD

A recent study investigated the efficacy of exclusive EN for 12 weeks in relieving inflammatory bowel strictures in 65 patients with CD. Among the 59 patients evaluated, 50 (84.7%) finished the EN treatment, whereas the other 9 patients (15.3%) were operated on because of progressive bowel obstruction. The intention-to-treat analysis revealed that 48 patients (81.4%) achieved symptomatic remission, 35 (53.8%) achieved radiologic remission, and 42 (64.6%) achieved clinical remission. The average luminal cross-sectional area at the site of stricture increased approximately 331% at week 12 suggesting that EN could effectively relieve inflammatory bowel stricture in CD [57]. The results of this study need further investigation regarding mainly the underlying mechanisms of this effect.

## 6. Diet Rich in Transforming Growth Factor-Beta in Patients with CD

During recent years, industry efforts, aiming to preserve the biological activity of some bioactive molecules in end-products, resulted in the production of food containing transforming growth factor-*β* (TGF-*β*), a polypeptide present in both human and bovine milk. TGF-*β* plays a critical role in the development of tolerance, in the prevention of autoimmunity, and in anti-inflammatory responses. It is, also, a potent inhibitor of intestinal epithelial cell growth and a stimulator of intestinal epithelial cell differentiation.

Modulen IBD is a polymeric diet with casein as its protein source, which is rich in TGF-*α*. The protein content is 14%, the carbohydrate content is 44%, and the fat content is 42%. It is lactose-free with glucose polymer and sucrose as the carbohydrate source. Its lipid content is made up of milk fat (55.6%), corn oil (13.9%), and medium chain triglycerides (26.1%). The osmolarity of feed is 312 mosm/L. It has been formulated to contain adequate amounts of vitamins, minerals, and trace elements. The patient must dilute 50 g of Modulen IBD powder in 210 mL of fresh water and consume the final product in about half an hour. Each meal offers more than 210 kcal.

In a relevant study [58], 29 adult patients with active CD received Modulen IBD as an exclusive diet for 4 weeks at a dose of 50 g × 5/d. Patients continued to be on their regular maintenance treatment. Clinical improvement was noticed in 69% of the patients. All nutritional parameters improved. Patients stopped losing weight and the score of general well-being increased. No change of the situation or worsening was noticed in 9 patients (31%) suggesting that this diet is effective in inducing remission in a proportion of adult patients with mild or moderately active CD. The almost complete absence of side effects makes this therapeutic modality quite attractive in patients suffering from mild to moderate flare-up of CD.

There is considerable speculation concerning the mode of action of Modulen IBD. In CD endogenous healing pathways mediated by TGF-*β* are inhibited because mucosal inflammatory cells express Smad7, the endogenous intracellular inhibitor of TGF-*α* signalling [59]. It seems unlikely that enteral feeds containing TFG-*β* exert their therapeutic effect by means of direct anti-inflammatory effects, although TGF-*β* may promote mucosal healing in synergy with changes in mucosal bacterial populations as a result of the change in the diet. Antigen exclusion and changes in bacterial flora seem to be the most important [60]. An important anti-inflammatory effect of TGF-*β* is the promotion and generation of FOXP3-positive regulatory T cells in the intestinal compartment [61].

Clinical response to Modulen IBD is associated with mucosal healing and downregulation of mucosal proinflammatory cytokine mRNA in both the terminal ileum and the colon. Experimental observations suggest that Modulen IBD supplementation could provide significant protection against weight loss, hypoalbuminemia, acidosis, and GI damage in a rat model [62].

Regarding the long-term efficacy of this special diet in maintaining remission, a group of investigators compared the results of the administration of Modulen IBD with those of mesalazine in a group of patients with CD in remission. Patients were randomly assigned to receive either two meals (2 × 50 g) of Modulen IBD plus two regular meals per day (43 patients) or mesalazine (800 mg three times a day) (40 patients) for six months. At the end of trial, 25 patients in the Modulen arm (69%) continued to be in remission compared with 18 (60%) receiving mesalazine (no significant differences). The mean time from remission to relapse was 103 days for those treated with mesalazine and 123 days for those treated with Modulen (no significant differences) [63]. An interesting finding of this study was the increase in the levels of HDL and the decrease in the levels of LDL lipoproteins in patients receiving Modulen IBD. The role of dysfunctional HDL in cytokine induction and inflammation seems to be quite important, as HDL can modulate LDL oxidation and LDL-induced cytokine production and inflammation [64]. Dysfunctional HDL has been identified in animal models and humans with chronic inflammatory diseases. Evidence suggests that the anti-inflammatory properties of HDL may be at least as important as the levels of HDL-cholesterol.

The pathophysiological consequences of Modulen IBD administration need further exploration, as the levels of HDL and LDL before and after treatment of IBD patients could be a useful index of inflammatory activity. Larger studies, especially in conjunction with other drugs including biologic agents, are needed in order to confirm the benefits derived from the administration of this kind of diet.

## 7. Enteral Nutrition in Patients with Ulcerative Colitis

Unlike CD, EN in patients with active UC has not been adequately studied. One prospective randomised trial compared the efficacy of total EN and total parenteral nutrition as an adjunct therapy in patients with acute severe UC on intensive corticosteroid therapy. After a 48 h steroid therapy, patients were randomized to receive polymeric total EN (*n* = 22) or TPN (*n* = 20). Remission rates and the need for colectomy were similar in the two groups. No significant change in anthropometric parameters was observed in either group. However, the 16.7% median increase in serum albumin in the EN group was significantly greater than 4.6% in the TPN group [65].

Enteral feeding with a polymeric formula following a 48 h intensive medical therapy was applied in 17 patients with severe flare-up of UC [66]. The formula concentration and volume were increased daily. In 14 of 17 patients EN was well tolerated, attaining more than 80% of the caloric requirements by day 4 in 11 patients. Prealbumin levels improved significantly. Although albumin and other nutritional parameters did not significantly improve in this study, the increase in prealbumin levels indicates a favourable anabolic effect. EN seems to be safe and nutritionally adequate in patients with severe attacks of UC.

The available data concerning the role of EN in patients with active UC are inadequate. Additional studies including larger cohorts of patients must be performed.

## 8. General Nutritional Suggestions for Patients with IBD

A normal diet should be suggested for all patients with IBD during remission, as maintenance of good nutritional status and replacement of losses represent the main targets of an effective treatment. Therefore, we must encourage our patients to consume normal diet, if their general health status allows it. Avoidance of milk and milk products during the exacerbations of the disease or in patients having lactase deficiency seems to represent a reasonable advice. In case of lactose intolerance, substitution of milk by other fermented products (especially “feta” cheese) or calcium-enriched soya-based products is recommended.

In case of intestinal stenosis causing incomplete bowel obstruction we can advise restrictions of fiber consumption. Fiber restriction could also be suggested in patients with irritable bowel syndrome coexisting with IBD. There are no data suggesting avoidance of fiber in the absence of enteric stenosis in patients with CD, although this kind of food could temporarily be restricted from the diet [67].

Patients with extensive small bowel involvement or resection should reduce the consumption of fat and those with extensive disease or terminal ileum resection with intact colon must avoid foods rich in oxalic salts and fat in order to reduce the possibility of nephrolithiasis. The consumption of calcium and/or cholestyramine should be adopted in these cases [68, 69].

Iron and folic acid deficiencies should be routinely monitored in patients with IBD due to their high occurrence. The measurement of transferring levels along with levels of ferritin could help us to distinguish between iron deficiency anaemia, anaemia due to chronic illness, and anaemia due to both situations. In cases of iron depletion, the IV administration is a reasonable way because iron administered by mouth can cause oxidative stress possibly resulting in exacerbation of the underlying IBD [14]. The resection or involvement of the terminal ileum in CD requires vitamin B12 supplementation* via* the parental route.

Patients must be encouraged to consume food rich in calcium at a dose of 1000 mg daily, as well as 400–800 IU vitamin D in order to avoid the appearance of osteoporosis. Calcium and vitamin D3 supplements are also required during treatments with systemic steroids [70].

Effective nutritional balance could be a complicated task requiring the cooperation of nutritionists and dieticians. Mutual trust among patients, their families, and health professionals is vital to ensure sufficient nutritional compliance required by this devastating chronic illness.

## 9. Conclusions

The available data indicate that EN, apart from restoring the impaired nutritional status, could also modulate intestinal immune responses, thus positively affecting the inflammatory bowel processes. EN could be characterized as either a supporting or primary therapy aiming to induce and maintain remission. EN might be used in CD patients when corticosteroid therapy is not possible.

It is important not to underestimate the role of nutrition as supportive care in patients with CD, despite the relatively few data supporting its use as a sole primary therapy to induce remission. Further well-designed large trials are necessary to support current knowledge on EN including long-term benefits. Improved evidence to support the role of dietary interventions for inducing and maintaining clinical remission could lead to a reduced need for drug therapy with its associated risk for adverse effects.

## Figures and Tables

**Table 1:** Causes of malnutrition in patients with IBD [7–9, 11–14].

Decrease in oral intake	(i) Restrictive diets (ii) Therapeutic fasting (iii) By the disease itself due to diarrhea, abdominal pain, nausea, and vomiting [9] (iv) Alteration in taste due to drugs, vitamin and mineral deficiencies, and proinflammatory mediators [7] (v) Anorexigenous effect of proinflammatory cytokines [7]

Gastrointestinal losses	(i) Diarrhea (ii) Rectorrhagia/hematochezia (iii) Loss of mucus and electrolytes (iv) Protein-losing enteropathy

Metabolic disorders	(i) Increase in resting energy expenditure due to inflammation, fever, and sepsis (ii) Enhanced fat oxidation

Increase in nutritional requirements	(i) Inflammatory states [7] (ii) Increased basal oxidative metabolism (iii) Infectious complications (iv) Postsurgery

Drug interaction	(i) Corticosteroids and calcium reabsorption (ii) Corticosteroids and protein catabolism (iii) Salazopyrin and folates [11] (iv) Methotrexate and folates [11] (v) Cholestyramine and liposoluble vitamins [11] (vi) Antimicrobials and vitamin K [12, 13] (vii) Antisecretors and iron [14]

Poor absorption of nutrients	(i) Reduction of the absorptive surface due to intestinal resection and enteric fistulas [8] (ii) Blind loops and bacterial overgrowth (iii) Poor absorption of bile salts in ileitis or resection [8] (iv) Mucosal inflammation

**Table 2:** Mechanisms of action of enteral nutrition in patients with IBD [15–18].

Sequestration of intraluminal antigens	
Modulation of the immune response of the bowel [15]	
Downregulation of proinflammatory cytokines	
Restore of the antioxidant status	
Anti-inflammatory effects [15]	
Alteration in the uptake of polyunsaturated fatty acids (n-6/n-3 fatty acids) [17]	
Enhancement of the restoration/function of the intestinal mucosal barrier (intestinal permeability) [18]	
Regulation of the intestinal microflora	
Regulation of the intestinal motility	
Promotion of epithelial healing	
Regulation of the bile-pancreatic secretions	
Sequestration of nutritional particles	
Improvement of nutritional status [16]	
Amelioration of the mesenteric adipose tissue hypertrophy [16]	

**Table 3:** Enteral nutrition (elemental and polymeric diet) on induction of remission in patients with Crohn's disease.

Author/reference	Number of patients	Diets compared	Outcomes/measurements	Results	Conclusion
Greenberg et al. [28]	51	TPN versus formula diet via NG versus partial parenteral and oral food	Relapse rates, weight, albumin, arm circumference, and triceps skinfold thickness	Clinical remission in 71% of parenteral group, 60% of partial parenteral group, and 58% defined formula group	In patients with active CD bowel rest is not a major factor in achieving a remission during nutritional support and did not influence outcome during one-year follow-up

Jones [29]	36	TPN versus elemental for induction of remission in CD	CDAI, ESR, and serum albumin	Both were successful with no significant differences. Elemental diet is cheaper, safer, and simpler	The use of EN followed by a personal food exclusion diet is an effective long-term therapeutic strategy for CD

Esaki et al. [30]	145	Enteral nutrition versus nonenteral (elemental or polymeric) nutrition	Rate of relapse based on CDAI scores	The rate of recurrence was higher in the nonenteral nutrition group than in the EN group	Among patients with CD under maintenance EN, the risk of recurrence differs according to the disease type and location. EN alone is insufficient for patients with penetrating type or with colonic involvement

**(a):** 

Author/reference	Total number of patients	Treatment's duration	Short-term remission		Long-term remission		Conclusion
			Non-ED (rate of remission)	ED (rate of remission)	Non-ED (rate of remission)	ED (rate of remission)	
Rigaud et al. [31]	30	6 weeks (short-term) 4 more weeks (long-term)	11/15 (73%)	10/15 (67%)	4/15 (27%)	3/15 (2%)	EN (non-ED or ED) is an efficient therapy for active CD. EN does not influence the long term outcome

Verma et al. [32]	21	6 weeks	6/11 (55%)	8/10 (80%)	—	—	EN is effective in treatment of active CD Polymeric and elemental diets are equally effective

Giaffer et al. [33]	30	4 weeks	5/14 (36%)	12/16 (75%)	—	—	ED offers significantly better short-term results compared to polymeric (non-elemental) diet in patients with acute exacerbations of CD

Non-ED = non-elemental diet, ED = elemental diet.

**(b):** 

Author/reference	Total patients' number	Treatment's duration	Short-term remission		Long-term remission		Conclusion
			Steroids (remission rate)	Enteral (^*^) nutrition (remission rate)	Steroids (remission rate)	Enteral (^*^) nutrition (remission rate)	
Morain et al. [25]	21	4 weeks (short-term) and 12 weeks (long-term)	8/10 (80%)	9/11 (82%)	7/10 (70%)	8/11 (73%)	EN is effective as CSs in inducing remission

Malchow et al. [24]	95	6 weeks	32/44 (73%)	21/51 (41%)	—	—	Drug combination was superior to EN in short-term remission

Lochs et al. [23]	107	6 weeks	41/52 (79%)	29/55 (53%)	—	—	EN is less effective than a combination of CSs and sulfasalazine in short-term remission

Gorard et al. [35]	33	12 weeks	17/20 (85%)	10/13 (77%)	6/19 (32%)	1/11 (9%)	EN is equally effective in the short term as CSs in CD The relapse rate after EN was greater than after treatment with CSs

Gonzalez-Huix et al. [37]	32	4 weeks (short-term) 56 weeks (long-term)	15/17 (88%)	12/15 (81%)	7/17 (41%)	10/15 (67%)	EN is as safe and effective as CSs in inducing short-term remission in active CD

Lindor et al. [41]	19	4 weeks	7/10 (70%)	3/9 (33%)	—	—	The often poorly tolerated EN should not be considered as a substitute for standard therapy with CSs in CD

^*^Enteral nutrition (elemental or polymeric).

**Table 5:** Prospective studies of enteral nutrition as maintenance therapy.

Author/reference	Enteral versus control (*n*)	Study characteristics	Duration of treatment (months)	Results (rate of recurrence)	Conclusion
Yamamoto et al. [46]	20/20	Prospective CD remission elemental overnight	12	5/20 versus 13/20	EN therapy reduces the incidence of postoperative CD recurrence

Yamamoto et al. [45]	20/20	Prospective postoperative elemental overnight	12	1/20 versus 7/20	Long-term EN in patients with quiescent CD improves clinical and endoscopic disease activities and the mucosal inflammatory cytokine levels

Takagi et al. [44]	26 (elemental diet group) 25 (free diet group)	Prospective CD remission elemental diet	24	35% versus 64%	A half elemental diet is a promising maintenance therapy for CD

Esaki et al. [49]	21/18	Prospective postoperative 1200 kcal/day versus 1200 kcal/day enteral	6–83	11/24 versus 12/16	EN could prevent the postoperative recurrence of terminal ileum CD. Patients with the penetrating type and those who do not have active lesions in the terminal ileum could receive EN after surgery

Verma et al. [43]	21 on EN in addition to normal diet versus 18 pts on normal, unrestricted diet	Prospective CD remission oral nutritional supplements	12	On an intention-to-treat basis, 10/21 (48%) remained in remission compared to 4/18 (22%) patients in Group 2, (*P* < 0.0003)	Nutritional supplementation is safe, well tolerated, and effective in the long-term management of patients with quiescent CD

Harries et al. [50]	28 malnourished patients with CD	Prospective crossover for 2 months oral nutritional supplements	4	Disease activity, nutritional status	Enteral supplementation can be managed successfully at home and may improve nutrition and disease activity
